# DAF-Aided ISAC Spatial Scattering Modulation for Multi-Hop V2V Networks

**DOI:** 10.3390/s25196189

**Published:** 2025-10-06

**Authors:** Yajun Fan, Jiaqi Wu, Yabo Guo, Jing Yang, Le Zhao, Wencai Yan, Shangjun Yang, Haihua Ma, Chunhua Zhu

**Affiliations:** 1 The Key Laboratory of Grain Information Processing and Control (Henan University of Technology), Ministry of Education, Zhengzhou 450001, China; yjfan@haut.edu.cn (Y.F.); ybguo@haut.edu.cn (Y.G.); yangjing@haut.edu.cn (J.Y.); yanwencai@haut.edu.cn (W.Y.); sjyang@haut.edu.cn (S.Y.); mahaihua@haut.edu.cn (H.M.); zhuchunhua@haut.edu.cn (C.Z.); 2 The College of Information Science and Engineering, Henan University of Technology, Zhengzhou 450001, China; jiaqiwu_5712@163.com; 3 School of Artificial Intelligence and Big Data, Henan University of Technology, Zhengzhou 450001, China

**Keywords:** integrated sensing and communications, multi-hop V2V networks, spatial scattering modulation, energy efficiency, detect-amplify-and-forward

## Abstract

Integrated sensing and communication (ISAC) has emerged as a transformative technology for intelligent transportation systems. Index modulation (IM), recognized for its high robustness and energy efficiency (EE), has been successfully incorporated into ISAC systems. However, most existing IM-based ISAC schemes overlook the spatial multiplexing potential of millimeter-wave channels and remain confined to single-hop vehicle-to-vehicle (V2V) setups, failing to address the challenges of energy consumption and noise accumulation in real-world multi-hop V2V networks with complex road topologies. To bridge this gap, we propose a spatial scattering modulation-based ISAC (ISAC-SSM) scheme and introduce it to multi-hop V2V networks. The proposed scheme leverages the sensed positioning information to select maximum signal-to-noise ratio relay vehicles and employs a detect-amplify-and-forward (DAF) protocol to mitigate noise propagation, while utilizing sensed angle data for Doppler compensation to enhance communication reliability. At each hop, the transmitter modulates index bits on the angular-domain spatial directions of scattering clusters, achieving higher EE. We initially derive a closed-form bit error rate expression and Chernoff upper bound for the proposed DAF ISAC-SSM under multi-hop V2V networks. Both theoretical analyses and Monte Carlo simulations have been made and demonstrate the superiority of DAF ISAC-SSM over existing alternatives in terms of EE and error performance. Specifically, in a two-hop network with 12 scattering clusters, compared with DAF ISAC-conventional spatial multiplexing, DAF ISAC-maximum beamforming, and DAF ISAC-random beamforming, the proposed DAF ISAC-SSM scheme can achieve a coding gain of 1.5 dB, 2 dB, and 4 dB, respectively. Moreover, it shows robust performance with less than a 1.5 dB error degradation under 0.018 Doppler shifts, thereby verifying its superiority in practical vehicular environments.

## 1. Introduction

With the rapid evolution of intelligent transportation systems, vehicles embedded with communication and sensing modules are emerging as dynamic network nodes in next-generation vehicular ad hoc networks [1]. Specifically, they rely on vehicle-to-everything (V2X) technology to exchange information with surrounding vehicles, infrastructure, pedestrians, and networks, achieving all-round network connectivity [2,3]. The convergence of heterogeneous sensing data from distributed vehicles forms a dense information network, where sensing supplies environmental information for communication, and in turn, communication enables the transmission of sensing data, thus promoting the application of integrated sensing and communication (ISAC) to V2X [4,5]. Existing studies on ISAC-V2X predominantly concentrate on the reliability of single-hop communication within dynamic mobility scenarios. However, they neglect several crucial challenges inherent to multi-hop V2V networks. These challenges include energy efficiency (EE) losses incurred due to redundant relaying in complex topologies, noise accumulation in traditional amplify-and-forward protocols, and signal degradation resulting from uncompensated Doppler shifts. Collectively, these issues impede the practical deployment of ISAC in vehicular networks.

Due to its better bit error rate (BER) performance and EE advantages, index modulation (IM) has been receiving a growing research interests in recent years. IM schemes convey the so-called index information in the activation status of transmit antennas [6] and subcarriers [7]. The concept of IM has been successfully incorporated into ISAC systems, enabling the co-existence of radar and communications. In [8], communication information was firstly embedded into the indices of the active antennas, i.e., generalized spatial modulation, and proposed a novel multiple-input multiple-output (MIMO) orthogonal frequency division multiplexing (OFDM) ISAC system. Specifically, the OFDM signals are transmitted via generalized spatial modulation. The shared subcarriers of OFDM enable high-speed communication, while dedicated subcarriers are utilized for sensing, achieving high angular resolution and antenna index estimation accuracy. In order to further increase the communication rates, the spatial modulation-based communication radar system was proposed in [9], in which the additional bits are conveyed by the combinations of transmit antenna units, and the antenna allocation pattern changes randomly between symbols, thus facilitating spatial flexibility.

IM can also be applied to the frequency domain for the integration of communication and sensing [10], i.e., subcarrier index modulation [7], conveying extra bits by the activated subcarriers indices. Recently, the work [11] proposed the multi-carrier agile joint radar communication (MAJoRCom) based on the spatial and the frequency agility. By leveraging the inherent spatial and spectral randomness of the carrier agile phased array radar (CAESAR), the MAJoRCom transmits the communication messages in the form of IM. Results indicate that transmission rates comparable to those of a separate communication module can be achieved without degrading radar performance. Different from CAESAR, existing vehicular radar systems typically adopt frequency modulated continuous wave (FMCW) radars. This motivates the FMCW-based joint radar–communications system [12], which conveys communication message in the combination of antenna selection, carrier selection, waveform permutation, and phase modulation. Performance analysis and simulation results demonstrate the advantages of communication and radar in terms of higher rates and the resolution capability, respectively.

To further enhance the radar sensing performance, the authors of [13] focused on applying the compressed sensing approach to acquire the range–velocity profile and proposed the IM-OFDM RadCom system. The authors of [14] focused power saved by IM onto activated subcarriers and proposed a low-complexity IM-OFDM ISAC algorithm by combining multiple sensing observations. This ensures IM-OFDM ISAC systems outperform OFDM ISAC systems in communication and sensing. However, the inherent unregulated auto-correlation of OFDM waveforms and their sensitivity to Doppler shifts restrict their widespread application in dynamic scenarios. Thus, the authors of [15] proposed an enhanced superposed IM-OFDM ISAC system. It converts the EE of IM-OFDM into a sensing-oriented signal on OFDM, enhancing sensing performance. Compared to the IM-based OFDM ISAC solution in [13,14], superposed IM-OFDM uses the sensed information to compensate for the Doppler effect over time-varying channels.

Recently, beam-domain index modulation in the millimeter-wave (mmWave) band has also gained attention for ISAC studies. In a more generalized version of IM-assisted mmWave systems, spatial path index modulation (SPIM)-assisted millimeter wave systems are studied [16,17,18,19]. Although [16,17] demonstrate that the SPIM-mmWave system outperform the current mmWave-MIMO systems in terms of higher spectral efficiency (SE) and better error performance under many common channel conditions, its application for ISAC scenarios is relatively scarce. For SPIM-based ISAC systems, [18] generated hybrid beamforming for radar targets and communication users simultaneously. Also in [19], a beam separation sensing algorithm was proposed to estimate radar and communication parameters for designing beamformers, thus improving SE.

From the above studies, we can see the advantages of ISAC-IM over the spatial domain, frequency domain, and beamspace domain. However, existing research mostly focuses on single-hop vehicle-to-vehicle (V2V) scenarios. Research on V2V scenarios remains relatively scarce. In particular, investigations into multi-hop networks are still in their preliminary stages [20,21]. Thus, we conduct research on ISAC-IM for multi-hop V2V networks to enhance reliability and EE. Conventional relay strategies include amplify-and-forward [22], detect-and-forward [23], and decode-and-forward [24]. However, amplify-and-forward amplifies both the signal and noise, causing noise accumulation. This makes it unreliable in low-signal-to-noise-ratio (SNR) conditions [25]. Decode-and-forward enhances reliability through information encoding, but it is complex, which renders it unable to meet the reliability requirements of low-latency, high-rate communication [26]. To address this issue, we propose a detect-amplify-and-forward (DAF) strategy to eliminate noise in communication and amplify the useful signal.

Owing to a high data rate demand in wireless communications, in this paper, we consider mmWave MIMO systems and propose DAF-based ISAC spatial scattering modulation (DAF ISAC-SSM) for multi-hop V2V networks. By leveraging the spatial directions of scattering clusters, DAF ISAC-SSM carries additional IM information at the transmitter and achieves a higher EE. Traditionally, the source vehicle (SV) will randomly set up a link and utilize beamforming to transmit information, relying on a global positioning system. For ISAC-SSM, instead, the positioning information sensed by radars help in the selection of the relay vehicle (RV) with the maximum SNR. Meanwhile, the RV adopts the proposed DAF strategy to avoid noise accumulation. In addition, we also utilize the sensed positioning information to counter Doppler, thus improving the reliability in V2V dynamic scenarios.

Compared with existing work, the main contributions of this study are summarized as follows:

- ISAC and SSM technology is introduced into multi-hop V2V networks to boost communication reliability and EE. DAF ISAC-SSM utilizes the spatial directivity of scattering clusters via one-RF chains, thereby boosting the EE performance. A Doppler compensation scheme is further designed with the sensed angel information.
- The multi-hop network is established in accordance with the sensed information by radar. Meanwhile, the DAF strategy is proposed at the RV to eliminate noise from the communication and amplify useful signals.
- The upper bound of the bit error rate (BER) of the proposed multi-hop DAF ISAC-SSM is derived based on the conditional pairwise error probability (CPEP) expression. Numerical Monte Carlo simulations have also been carried out to demonstrate the superiority of DAF ISAC-SSM over existing alternatives in terms of EE and error performance.

The reminder of this paper is organized as follows. Section 2 describes the system and channel models. Section 3 elaborates on the theoretical analysis for DAF ISAC-SSM. Simulations are presented in Section 4, followed by conclusion in Section 5.

*Notations*: A, a, and *a* stand for a matrix, a vector, and a scalar, respectively. $A^{*}\left(a^{*}\right)$ is the conjugate transpose of A(a). $A^{T}$ denotes the transpose operator of a vector. ${∥\cdot ∥}_{F}$ represents the Frobenius norm of a vector. $\left|\cdot \right|$ represents the absolute value of a scalar. Q(·) and δ(·) represent the Gaussian Q-function and the Dirac delta function, respectively. The list of acronyms are presented in Table 1.

## 2. System and Channel Models

**Figure 1:**
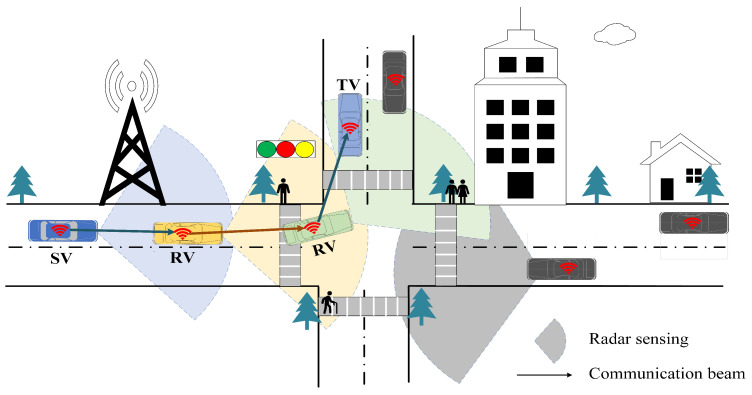
The schematic of the studied ISAC multi-hop V2V network.

We consider a multi-hop V2V networking in Figure 1, where each vehicle is equipped with communication devices for information exchange and radar devices for sensing surroundings, respectively. The notations relating to the DAF ISAC-SSM system are presented in Table 2.

### 2.1. Channel Model

Each vehicle is equipped with $N_{c,t}$ transmit antennas and $N_{c,r}$ receiver antennas for information exchange. In this paper, to characterize the finite scattering characteristics of mmWave propagation, we adopt the geometric Saleh–Valenzuela channel model [27]. Specifically, we utilize a simplified channel model, assuming that each scattering cluster contributes a single path to the channel [17]. Under this assumption, we define *n* as a certain hop and *l* as a specific link within that hop. Without loss of generality, each path has only one scattering cluster, and *I* is the total scattering clusters. According to [17], the channel matrix at (hop *n*, link *l*, and time instance *m*) can be expressed as

(1) $$H_{c,n,l,m}=\sqrt{\frac{N_{c,t}N_{c,r}}{I}}\sum_{i=1}^{I}\alpha_{n,l,i}a_{r}\left(\theta_{n,l,i}^{r}\right){a_{t}}^{*}\left(\theta_{n,l,i}^{t}\right)e^{jw_{n,l,i}m},$$

where $\alpha_{n,l,i}$ is the gain of the *i*-th scattering cluster. The normalized Doppler frequency shift is defined as $w_{n,l,i}=2\pi f_{c}vT_{s}\sin\left(\theta_{n,l,i}^{t}\right)/c_{v}$, with *v* being the relative velocity between two vehicles, $T_{s}$ being sampling time, $c_{v}$ being the speed of light, and $f_{c}$ being the carrier frequency. By defining the angle of arrival as $\theta_{n,l,i}^{r}$ and the angle of departure as $\theta_{n,l,i}^{t}$, the $N_{c,t}$- and $N_{c,r}$-dimensional array responses at the transmitter and receiver under the half-wavelength uniform linear arrays, namely $a_{t}\left(\theta_{n,l,i}^{t}\right)\in C^{N_{c,t}\times 1}$, and $a_{r}\left(\theta_{n,l,i}^{r}\right)\in C^{N_{c,r}\times 1}$, are given by

(2) $$\;\;\;\;\;a_{t}\left(\theta_{n,l,i}^{t}\right)=\frac{1}{\sqrt{N_{c,t}}}{\left[1,e^{j2\pi \psi_{n,l,i}^{t}},\ldots ,e^{j2\pi \psi_{n,l,i}^{t}\left(N_{c,t}-1\right)}\right]}^{T},$$

(3) $$\;\;\;a_{r}\left(\theta_{n,l,i}^{r}\right)=\frac{1}{\sqrt{N_{c,r}}}{\left[1,e^{j2\pi \psi_{n,l,i}^{r}},\ldots ,e^{j2\pi \psi_{n,l,i}^{r}\left(N_{c,r}-1\right)}\right]}^{T},$$

where $\psi_{n,l,i}^{t}\overset{\Delta }{=}\frac{d_{c,t}}{\lambda_{c}}\sin\left(\theta_{n,l,i}^{t}\right)$ and $\psi_{n,l,i}^{r}\overset{\Delta }{=}\frac{d_{c,r}}{\lambda_{c}}\sin\left(\theta_{n,l,i}^{r}\right)$ represent the phase difference for transmitter and receiver, respectively. Also, $\lambda_{c}$ is the carrier wavelength, and $d_{c,t}$ and $d_{c,r}$ denote the antenna spacing for transmitter and receiver, respectively.

Specifically, when $N_{c,t}$ and $N_{c,r}$ are sufficiently large, the beamwidth between the transmitter and receiver becomes narrower. Thus the interference between scattering clusters is restricted [17]. Taking $i_{1}$ and $i_{2}$ as examples to denote the transmit beams pointing to two different scattering clusters, and using the transmitted array response as an example, the inner product of the two array responses can be expressed as

(4) $$\begin{array}{cc} a_{t}{\left(\theta_{n,l,i_{1}}^{t}\right)}^{*}a_{t}\left(\theta_{n,l,i_{2}}^{t}\right) & =\frac{1-e^{j2\pi \left(\theta_{n,l,i_{1}}^{t}-\theta_{n,l,i_{2}}^{t}\right)N_{c,t}}}{N_{c,t}-N_{c,t}e^{j2\pi (\theta_{n,l,i_{1}}^{t}-\theta_{n,l,i_{2}}^{t})}}\\ \; & =\frac{\sin(\pi \left(\theta_{n,l,i_{1}}^{t}-\theta_{n,l,i_{2}}^{t}\right)N_{c,t})}{N_{c,t}\sin\left(\pi \left(\theta_{n,l,i_{1}}^{t}-\theta_{n,l,i_{2}}^{t}\right)\right)}e^{j2\pi \left(\theta_{n,l,i_{1}}^{t}-\theta_{n,l,i_{2}}^{t}\right)\left(N_{c,t}-1\right)}. \end{array}$$

Then we have

(5) $$\left|a_{t}{\left(\theta_{n,l,i_{1}}^{t}\right)}^{*}a_{t}\left(\theta_{n,l,i_{2}}^{t}\right)\right|=\frac{1}{N_{c,t}}\left|\frac{\sin(\pi \left(\theta_{n,l,i_{1}}^{t}-\theta_{n,l,i_{2}}^{t}\right)N_{c,t})}{\sin(\pi \left(\theta_{n,l,i_{1}}^{t}-\theta_{n,l,i_{2}}^{t}\right))}\right|.$$

Without loss of generality, it is assumed that all scatterers in DAF ISAC-SSM V2V communication are distributed between the transmitter and receiver channel, i.e., $\left(\theta_{n,l,i_{1}}^{t}-\theta_{n,l,i_{2}}^{t}\right)\in \left[-\pi /2,\pi /2\right]$ is satisfied. Therefore, we can get

(6) $$\left|\frac{\sin(\pi \left(\theta_{n,l,i_{1}}^{t}-\theta_{n,l,i_{2}}^{t}\right)N_{c,t})}{\sin(\pi \left(\theta_{n,l,i_{1}}^{t}-\theta_{n,l,i_{2}}^{t}\right))}\right|\neq 0,$$

when $N_{c,t}$ tends to infinity, Equation (5) can be simplified to

(7) $$\left|a_{t}{\left(\theta_{n,l,i_{1}}^{t}\right)}^{*}a_{t}\left(\theta_{n,l,i_{2}}^{t}\right)\right|=0.$$

Note that when the number of transmit and receive antennas are large, the resulting beam is very narrow. At this point, we consider that there is interference between the beams; the inner product of the array responses is expressed as [17]

(8) $$\left\{\begin{array}{c} a_{t}{\left(\theta_{n,l,i_{1}}^{t}\right)}^{*}a_{t}\left(\theta_{n,l,i_{2}}^{t}\right)\approx \delta \left(i_{1}-i_{2}\right)\\ a_{r}{\left(\theta_{n,l,i_{1}}^{r}\right)}^{*}a_{r}\left(\theta_{n,l,i_{2}}^{r}\right)\approx \delta \left(i_{1}-i_{2}\right) \end{array}\right..$$

The mentioned Equation (8) further implies that the interference among scattering clusters is constrained. Hence, within the context of this paper, we assume an exact orthogonality among all angles of arrival and angles of departure. Similar to [17], This assumption is utilized to simplify the theoretical computations in this paper.

### 2.2. Radar Model

The radar transmission power and the distance between the two vehicles at (link *l* and hop *n*) are denoted by $E_{r,n,l}$ and $R_{n,l}$, respectively. Similar to [28], the SNR is calculated as

(9) $$\gamma_{r,n,l}=\frac{E_{r,n,l}N_{r,r}G_{t}G_{r}\Omega \lambda_{r}^{2}}{{\left(4\pi \right)}^{3}\left(\eta_{r}+C+J\right)R_{n,l}^{4}},$$

where Ω represents the target’s cross-section of the radar detected target, and $\lambda_{r}$ is the carrier wavelength of the radar. The gains of the radar transmission antenna and reception antenna are denoted by $G_{t}$ and $G_{r}$, respectively. Additionally, consider a more realistic scenario that includes three types of noise: receiver noise $\eta_{r}$, cluster interference *C*, and interference from adjacent vehicles *J*. Particularly, the receiver noise can be represented as $\eta_{r}=kT_{0}FB_{r}$, where *k* is the Boltzmann’s constant, $T_{0}$ is the noise temperature, $B_{r}$ is the reception bandwidth, and *F* is the noise figure of the radar receiver, respectively. According to [29], the interference from adjacent vehicles can be represented as $J=\sum_{j}z^{2}E_{r,n,l}N_{r,r}G_{t}G_{r}\omega \lambda_{r}^{2}/{\left(4\pi \right)}^{3}R_{j}^{4}$, where *z* is the interference to the target vehicle from the transmission power of surrounding vehicles, satisfying 0≤z<1, and $R_{j}$ (in meters) represents the distance from the *j*-th neighboring vehicle. Similar to [28], the values used in the simulations are as follows: $k=1.38\times 10^{-23}$, $T_{0}=290$, F=4.5, $C=1.2153\times 10^{-11}$, and z=0.01.

The threshold SNR at the radar receiver is set to be a certain value $\gamma_{r,th}$. To ensure effective radar detection, the threshold SNR must satisfy $\gamma_{r,n,l}\geq \gamma_{r,th}$.

### 2.3. Communications Model

**Figure 2:**
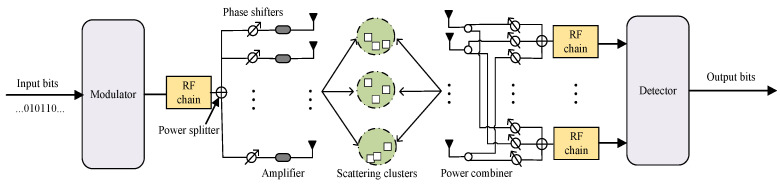
The schematic of the proposed DAF ISAC-SSM.

The detail system model of the DAF ISAC-SSM system is shown in the Figure 2, encompassing the transmission and the detection parts.

(1) DAF ISAC-SSM Transmission: The transmitter indexes and activates the corresponding scattering clusters. The information bits are divided into two parts for transmission. The first part, comprising $\log_{2}\left(M\right)$ bits, are modulated into symbols x∈φ through the use of an *M*-ary quadrature amplitude modulation (QAM) constellation, with φ representing the set of modulation symbols.

To transmit the symbols of the first part, $\log_{2}\left(I_{s}\right)$ bits of the information bits are utilized to form a beam directed towards the receiver. We sort the gains $\left|\alpha_{n,l,i}\right|$ of the scattering paths constituting the channel in descending order. Without loss of generality, $\left|\alpha_{n,l,1}\right|>\left|\alpha_{n,l,2}\right|>\cdots \left|\alpha_{n,l,I}\right|$. In each time slot, the top $I_{s}$ scattering paths having the highest path gains are chosen as candidate scattering paths [17]. One of the $I_{s}$ ($I>I_{s}$) candidate scattering clusters is selected as the transmission direction. Therefore, the achievable SE of the proposed DAF ISAC-SSM system is

(10) $$\eta_{SE}=\log_{2}\left(M\right)+\log_{2}\left(I_{s}\right).$$

Let *x* be the transmitted symbol to the desired relay vehicle. For transmit information, beamforming at (hop *n*; link *l*) is carried out on the antennas as follows

(11) $$p_{n,l}=\frac{1}{\sqrt{N_{c,t}}}{\left[1,e^{j2\pi \psi_{n,l,g}^{t}},\ldots ,e^{j2\pi \psi_{n,l,g}^{t}\left(N_{c,t}-1\right)}\right]}^{T},$$

where $\psi_{n,l,g}$ is the phase difference of the top $I_{s}$ scattering paths for transmitter, i.e., $g\in (1,\ldots ,I_{s})$.

During the first hop, the source vehicle transmits the signal to the relay vehicle. Notably, the source vehicle does not engage in signal-forwarding activities. Consequently, the received signal at the relay vehicle during the first hop (at hop *n*, link *l*, and time instant *m*) can be derived as

(12) $$y_{c,n,l,m}=\sqrt{\frac{E_{c,n,l}{\lambda_{c}}^{2}}{{\left(4\pi R_{n,l}\right)}^{2}}}H_{c,n,l,m}p_{n,l}x+n_{n,l,m},$$

where $E_{c,n,l}$ represents the communication transmit power, and $n_{n,l,m}∼CN\left(0,N_{0}I_{N_{c,r}}\right)$ is an additive white Gaussian noise vector.

When the number of hops satisfies n≥2 [17,30], DAF is utilized to relay the signal to the subsequent hop. Specially, the relay first detects (demodulates and decodes) the received signal. Then, the relay then re-modulates the decoded bits into a new, clean signal and amplifies it for transmission to the next hop. Under these circumstances, the received signal can be expressed as

(13) $$y_{c,n,l,m}=a_{n,l}\sqrt{\frac{E_{c,n,l}{\lambda_{c}}^{2}}{{\left(4\pi R_{n,l}\right)}^{2}}}H_{c,n,l,m}p_{n,l}x+n_{n,l,m},$$

where $a_{n,l}$ is the amplification factor when adopting the DAF strategy, which is capable of eliminating noise accumulation while amplifying the amplitude of the useful signal. It can be specifically expressed as

(14) $$a_{n,l}=\sqrt{\frac{E_{c,n,l}}{E_{c,n+1,l}{\left(R_{n,l}\right)}^{-\varepsilon }+N_{0}}},$$

where ε is the path loss factor. For the convenience of research, in the subsequent introduction, let $A_{n,l}=a_{n,l}\sqrt{E_{c,n,l}{\lambda_{c}}^{2}/{\left(4\pi R_{n,l}\right)}^{2}}$.

It is worth noting that the implementation of the DAF protocol in multi-hop V2V networks presents three key challenges: (1) error propagation, where incorrect detection at relay nodes amplifies errors across hops, particularly detrimental in low-SNR regimes; (2) latency and processing overhead, as demodulation–remodulation operations at each relay introduce delays that may hinder ultra-low-latency safety applications; and (3) synchronization and channel dynamics, since accurate channel state information acquisition becomes challenging in high-mobility scenarios. To mitigate these issues, our proposed scheme leverages ISAC-derived side information (e.g., angle/Doppler estimates) to enable sensing-aided relay selection (optimizing SNR) and enhance synchronization robustness. While DAF inherently trades off latency for reliability, its core advantage lies in preventing noise accumulation across hops—making it superior to amplify-and-forward in long-range multi-hop scenarios. The integration of ISAC thus serves as a critical step toward practical DAF deployment in dynamic V2V environments.

The following example illustrates the DAF ISAC-SSM with four selected scattering clusters in a multi-hop V2V network. For the input data stream $b=[b_{1},b_{2},\cdots ]$, a subset of bits whose length corresponds to a length of $\eta_{SE}$ is extracted. The initial $\left[b_{1},b_{2}\right]$ bits are utilized for QAM modulation with a modulation order of M=4. In contrast, the subsequent $\left[b_{3},b_{4}\right]$ bits are employed to index the $I_{s}$ = 4 scattering clusters. The Table 3 depicts the transmission scheme in detail. Thus, for $b=\left[b_{1},b_{2},b_{3},b_{4}\right]=\left[0,0,0,0\right]$, Equation (13) becomes

(15) $$\begin{array}{cc} y_{c,n,l,m} & =A_{n,l}H_{c,n,l,m}a_{t}\left(\theta_{n,l,1}^{t}\right)x+n_{n,l,m}\\ \; & =A_{n,l}\sqrt{\frac{N_{c,t}N_{c,r}}{I}}a_{r}\left(\theta_{n,l,1}^{r}\right)\alpha_{n,l,1}\frac{\left(1+1j\right)}{\sqrt{2}}+n_{n,l,m}. \end{array}$$

(2) ISAC-SSM Detection: The receiver (Rx) signal $y_{c,n,l,m}$ is subjected to phase manipulation through shifters, combined by each RF chain, and subsequently down-converted. When the weights of the Rx phase shifters are specified $a_{r}\left(\theta_{n,l,g}^{r}\right)$, with $g\in (1,\cdots ,I_{s})$ corresponding to the transmission direction, the maximum SNR can be attained. Given the Rx’s inability to accurately anticipate the beamforming direction of the transmitter (Tx), multiple RF chains are employed at the Rx to generate beams that are oriented towards the candidate scattering clusters. Denoting $r_{1:I_{s}}$ as the phase shifter weights for steering towards the scattering cluster, we have

(16) $$r_{1:I_{s}}=\left[a_{r}\left(\theta_{n,l,1}^{t}\right),\ldots ,a_{r}\left(\theta_{n,l,I_{s}}^{t}\right)\right].$$

The signal after the RF chain can be represented as

(17) $$\begin{array}{c} {\bar{y}}_{c,n,l,m}={\left(r_{1:I_{s}}\right)}^{*}y_{c,n,l,m} \end{array}.$$

To recover the transmitted bit information, the maximum likelihood (ML) detector can be expressed as

(18) g^,x^=argming∈1,⋯Is,x∈φy¯c,n,l,m(g)−An,lar(θn,l,gr)*Hc,n,l,mpn,lx2,

where $\hat{g}$ is the detected transmission direction, and $\hat{x}$ is the detected transmission symbol.

Owing to the existence of radar, we utilize the perceived angle information for Doppler shift compensation. The received signal following compensation is expressed as

(19) $$\begin{array}{c} {\tilde{y}}_{c,n,l,m}=e^{-j2\pi f_{c}vT_{s}\left(\sin\left(\theta_{n,l,i}^{t}\right)+e_{n,l}\right)m/c_{v}}{\bar{y}}_{c,n,l,m} \end{array},$$

where $e_{n,l}$ represents the angle error resulting from imperfect radar detection. It is further modeled as a Gaussian distribution with a mean of zero and a variance of $\sigma_{w}^{2}$.

More specifically, the Doppler compensation scheme is fundamentally enabled by the tight integration of sensing and communication, where the normalized Doppler frequency shift $w_{n,l,i}=2\pi f_{c}vT_{s}\sin\left(\theta_{n,l,i}^{t}\right)/c_{v}$ directly couples the vehicle’s motion to the sensed angle of departure ($\theta_{n,l,i}^{t}$). This relationship underscores that accurate angle estimation is critical for quantifying and mitigating Doppler effects. The first-order compensator operates as a phase derotator, applying a conjugate phase shift $e^{-jw_{n,l,i}m}$ to the received signal symbol-by-symbol, thereby counteracting the linear phase drift induced by mobility. The compensation process forms a closed-loop cycle: (1) radar sensing extracts real-time angle information from echo signals, (2) the Doppler shift $w_{n,l,i}$ is computed from the sensed angle, and (3) the derived phase correction is applied to the data stream. To address practical imperfections, the model explicitly incorporates an angle error term $e_{n,l}∼CN\left(0,\sigma_{w}^{2}\right)$, allowing rigorous quantification of the compensator’s robustness to sensing inaccuracies. The reliability of angular data in dynamic environments stems from its direct acquisition via the radar’s signal processing chain, which provides high refresh rates (millisecond-scale) and sub-degree spatial precision, ensuring timely adaptation to vehicular dynamics. This seamless fusion of sensing and communication is pivotal for enhancing reliability in high-mobility V2V scenarios.

When the hop count exceeds two, we denote the static version of $H_{c,n,l,m}$ as $H_{c,n,l}$ (where $w_{n,l,i}=0$). By substituting Equation (17) into Equation (19), we are able to derive that the signal power is $A_{n,l}^{2}{∥{\left(r_{1:I_{s}}\right)}^{*}H_{c,n,l}p_{n,l}∥}_{F}^{2}$, while the noise power is $∥{\left(r_{1:I_{s}}\right)}^{*}n_{n,l,m}{∥}_{F}^{2}$. The instantaneous SNR of the compensated received signal, which is defined as the ratio of the signal power to the noise power, can be explicitly expressed as follows:

(20) $$\gamma_{c,n,l}=A_{n,l}^{2}\frac{∥{\left(r_{1:I_{s}}\right)}^{*}H_{c,n,l}p_{n,l}{∥}_{F}^{2}}{∥{\left(r_{1:I_{s}}\right)}^{*}n_{n,l,m}{∥}_{F}^{2}}.$$

The threshold SNR at the communication receiver is set to be a certain value $\gamma_{c,th}$. To ensure effective communication, the threshold SNR must satisfy $\gamma_{c,n,l}\geq \gamma_{c,th}$.

### 2.4. Network Construction

In multi-hop ISAC-SSM V2V communication, the source vehicle determines the distance through the calculation of the time difference between the transmitted and received radar signals. Simultaneously, it gauges the angle by measuring the phase difference among the echo signals. By leveraging the sensed information, vehicles can obtain detailed real-time motion status information of surrounding vehicles, i.e., distance and angle, which helps build multi-hop networks according to the following steps:

(1) Given that the radar’s threshold SNR is defined as $\gamma_{r,th}$ and its maximal sensing power is defined as $E_{r,max}$, the maximum detection range of the radar can be derived as
  (21) $$R_{r,max}=\sqrt[{4}]{\frac{E_{r,max}N_{r,r}N_{r,t}G_{t}G_{r}\Omega {\lambda_{r}}^{2}}{{\left(4\pi \right)}^{3}\left(\eta_{r}+C+J\right)\gamma_{r,th}}}.$$
  The radar detection angle can be expressed as
  (22) $$\Theta_{r,max}=\arcsin\left(\frac{\varphi_{r}\cdot \lambda_{r}}{2\pi \cdot d_{r,r}}\right),$$
  where $d_{r,r}$ is the spacing between radar antennas, $\lambda_{r}$ is the carrier’s wavelength, and $\varphi_{r}$ is phase difference, respectively.
(2) Given that the communication (COMM)’s threshold SNR is defined as $\gamma_{c,th}$ and its maximal COMM power is defined as $E_{c,max}$, the maximum COMM range is
  (23) $$R_{c,max}=\sqrt[{2}]{\frac{E_{c,max}N_{c,r}N_{c,t}\lambda_{c}^{2}}{{\left(4\pi \right)}^{2}N_{0}\gamma_{c,th}I}}.$$
(3) At each hop, the RV is chosen from within both the radar detection range and the COMM range.
(4) The SNR of each candidate relay vehicle is measured. The vehicle with the maximum SNR is designated as the relay node, ensuring the establishment of robust and reliable COMM links within the network.
(5) Repeat Step-3 and Step-4 until the candidate RV becoming the target vehicle.

## 3. Analysis for DAF ISAC-SSM

This section builds upon the multi-hop DAF ISAC-V2V network model and substantiates the advantages of the proposed scheme from two aspects: energy efficiency and error performance.

### 3.1. Energy Efficiency

The above analysis derives the BER to evaluate the reliability of the system. Subsequently, EE is introduced to further evaluate the merits of DAF ISAC-SSM. Specifically, EE is defined as the ratio of SE to the total transmit power. In accordance with [31], the EE of DAF ISAC-SSM can be formulated

(24) $$\begin{array}{cc} \eta_{EE} & =\frac{\eta_{SE}}{P_{T}+P_{H}}\\ \; & =\frac{\log_{2}\left(M\right)+\log_{2}\left(I_{s}\right)}{P_{T}+P_{RF}+N_{c,t}P_{A}+N_{c,t}P_{ps}+P_{sp}}, \end{array}$$

where $P_{T}$ is the transmission power, and $P_{H}$ denotes the power consumed by hardware architecture. $P_{RF}$, $P_{A}$, $P_{ps}$, $P_{sp}$, and $N_{RF}$ are the power consumed by the RF chain, amplifier, phase shifter, power splitter, and the number of activated RF chains, respectively. The detailed definitions along with the power consumption related to $P_{H}$ are presented in Table 4.

### 3.2. Error Performance

Assume that the network consists of *N* viable hops. Given the independence among all network hops, the BER expression of DAF ISAC-SSM can be approximated as [32]

(25) $$\begin{array}{c} P=1-\prod_{n=1}^{N}\left(1-P_{n}\left(\alpha_{n,l,1},\ldots ,\alpha_{n,l,I_{s}}\right)\right) \end{array},$$

where $P_{n}\left(\alpha_{n,l,1},\ldots ,\alpha_{n,l,I_{s}}\right)$ for n∈(1,…,N) denotes the BER of the *n*-hop and can be expressed as [33,34]

(26) $$\begin{array}{c} \;\;P_{n}\left(\alpha_{n,l,1},\cdots ,\alpha_{n,l,I_{s}}\right)\leq \frac{1}{N_{B}N_{b}\left(g,x\right)}\sum_{g,x}\sum_{\hat{g},\hat{x}}\\ \;\;P_{n}(\{\left\{g,x\right\}\;\to \;\left\{\hat{g},\hat{x}s,\alpha_{n,l,I_{s}}\right)E_{b}\left(\left\{g,x\right\}\;\to \;\left\{\hat{g},\hat{x}\right\}\right) \end{array},$$

where $N_{B}$ is the total number of bits transmitted each time. The CPEP is denoted as $P_{n}\left(\left\{\left\{g,x\right\}\to \left\{\hat{g},\hat{x}\right\}\right\}|\alpha_{n,l,1},\cdots ,\alpha_{n,l,I_{s}}\right)$. $N_{b}\left(g,x\right)$ is the total number of possible realizations of *g* and *x*. $E_{b}\left(\left\{g,x\right\}\to \left\{\hat{g},\hat{x}\right\}\right)$ denotes the number of erroneous bits when *g* and *x* are transmitted but $\hat{g}$ and $\hat{x}$ are detected. The BER is affected not only by the CPEP but also by the number of scatters and the modulation order.

Assuming that the gains of the candidate scattering paths are known such that $\left|\alpha_{n,l,1}\right|>\left|\alpha_{n,l,2}\right|>\cdots \left|\alpha_{n,l,I_{s}}\right|$, denote the indices of the correctly detected scattering clusters and transmitted symbols as *g* and *x*, respectively. The detection process can be partitioned into two parts: the candidate scattering cluster index and the transmitted symbol. The derivation process can be classified into two scenarios: the case of correct detection of the scattering cluster index $g=\hat{g}$ and the case of incorrect detection of the scattering cluster index $g\neq \hat{g}$. Taking the *n*-th hop as an example, we then present the expression of the *n*-th hop CPEP for the proposed DAF ISAC-SSM scheme.

**Proposition** **1.** *When the scattering cluster index is accurately detected, the bit errors arise from symbol detection, i.e, $x\neq \hat{x}$. The upper bound of the hop-n CPEP is derived as follows*

(27) $$\begin{array}{c} P_{n}\left(\left\{g,x\right\}\to \left\{g,\hat{x}\right\}|\alpha_{n,l,1},\cdots ,\alpha_{n,l,I_{s}}\right)\leq \frac{1}{2}e^{-\frac{{{\bar{A}}_{n,l}^{2}\left|\alpha_{n,l,g}\right|}^{2}{\left|\left(x-\hat{x}\right)\right|}^{2}}{4N_{0}}} \end{array},$$

*where ${\bar{A}}_{n,l}=A_{n,l}\sqrt{N_{c,t}N_{c,r}/I}$, and $N_{0}$ represents the noise variance.*

**Proof.**  The detailed derivation can be found in Appendix A. □

**Proposition** **2.** *When the scatter index detection is erroneous, the symbol detection may either be correct, denoted as $x=\hat{x}$, or incorrect, denoted as $x\neq \hat{x}$. The hop-n CPEP is derived as follows*

(28) $$\begin{array}{c} P_{n}\left(\left\{g,x\right\}\to \left\{\hat{g},\hat{x}\right\}|\alpha_{n,l,1},\cdots ,\alpha_{n,l,I_{s}}\right)=\frac{1}{2}e^{\frac{-{\bar{A}}_{n,l}^{2}{\left|\alpha_{n,l,\hat{g}}\right|}^{2}{\left|\hat{x}\right|}^{2}}{2N_{0}}} \end{array}.$$

**Proof.**  The detailed derivation can be found in Appendix B. □

## 4. Simulations Results

In this section, we conduct performance comparison simulations of our proposed DAF ISAC-SSM against DAF ISAC-conventional spatial multiplexing (CSM), DAF ISAC-maximum beamforming (MBF), and DAF ISAC-random beamforming (RBF), focusing on EE and BER. Among the aforementioned comparison system, CSM employs the top $I_{s}$ scattering paths for transmission without scattering-based IM. MBF utilizes the scattering path having the largest gain for transmission, while RBF randomly selects one from the $I_{s}$ clusters. The system and channel parameters pertinent to the simulation are furnished in Table 5. Without other specifications, the distance between the SV and TV is fixed at 150 m, and the distance between different lanes is 4 m [28].

### 4.1. EE Comparison

**Figure 3:**
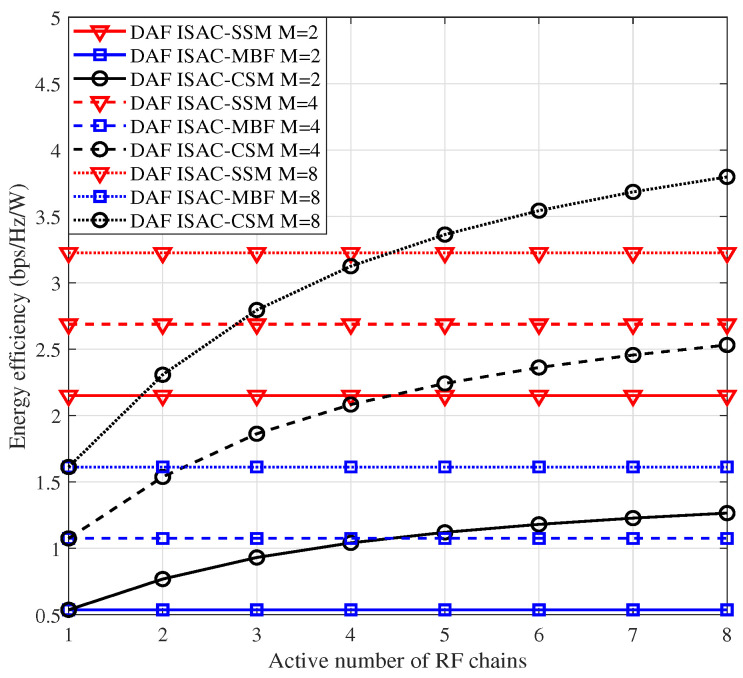
EE comparisons among DAF ISAC-SSM, DAF ISAC-MBF, and DAF ISAC-CSM.

In Figure 3, we compare the EE performance of DAF ISAC-SSM, DAF ISAC-CSM, and DAF ISAC-MBF. Given that DAF ISAC-MBF utilizes only a single RF chain, the SE is $\eta_{SE\_MBF}=\log_{2}\left(M\right)$, and the hardware loss power $P_{H}$ is equal to DAF ISAC-SSM. Regarding DAF ISAC-CSM, $\eta_{SE\_CSM}=N_{RF}\log_{2}\left(M\right)$, with $N_{RF}$ being the activated RF chain. Similar to [31], DAF ISAC-CSM adopts full-phased array based hybrid precoding, thereby leading to a hardware power consumption formulated as $P_{H}=N_{RF}P_{RF}+N_{c,t}P_{A}+N_{c,t}N_{RF}P_{ps}+N_{RF}P_{sp}+N_{c,t}P_{co}$. The transmission power $P_{T}$ is set to 1 W. These comparisons are conducted under the scenario of $I_{s}=8$, considering three different modulation orders, M=2, M=4, and M=8, respectively. From Figure 3, we can conclude the following observations:

- Given that only one RF chain is activated in both DAF ISAC-SSM and DAF ISAC-MBF, their EE remains unaffected by the number of active RF chains and thus remains constant. Moreover, DAF ISAC-SSM consistently outperforms DAF ISAC-MBF because it can exploit a higher SE.
- When the modulation order M≤4, DAF ISAC-SSM is always superior to DAF ISAC-CSM. This is because although an increasing number of RF chains boost a higher SE, DAF ISAC-CSM concurrently engenders additional power consumption. When *M* = 8, DAF ISAC-SSM becomes inferior to DAF ISAC-CSM for $N_{RF}>4$. The reason is that a higher SE can compensate for the extra power consumption.

### 4.2. BER in Time-Invariant Channel

**Figure 4:**
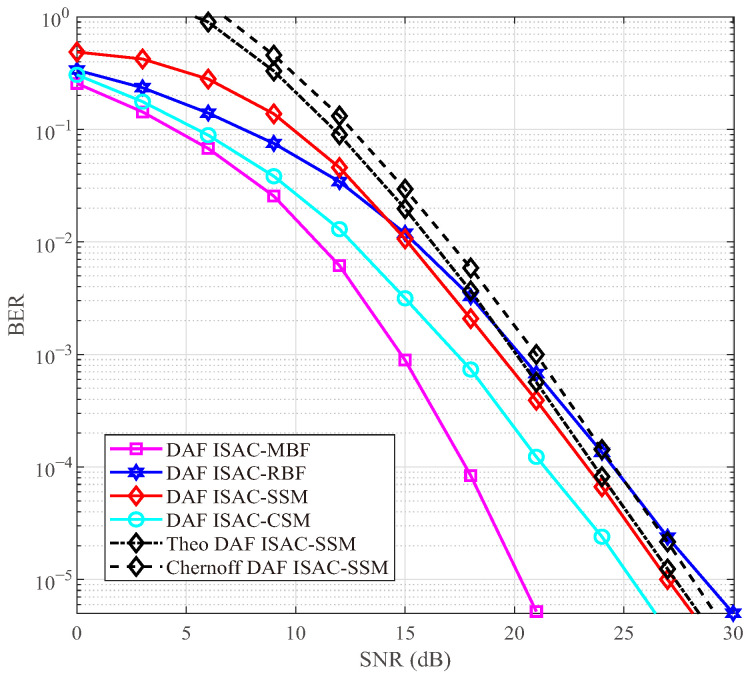
BER comparisons under 2-hop network with I=6.

**Figure 5:**
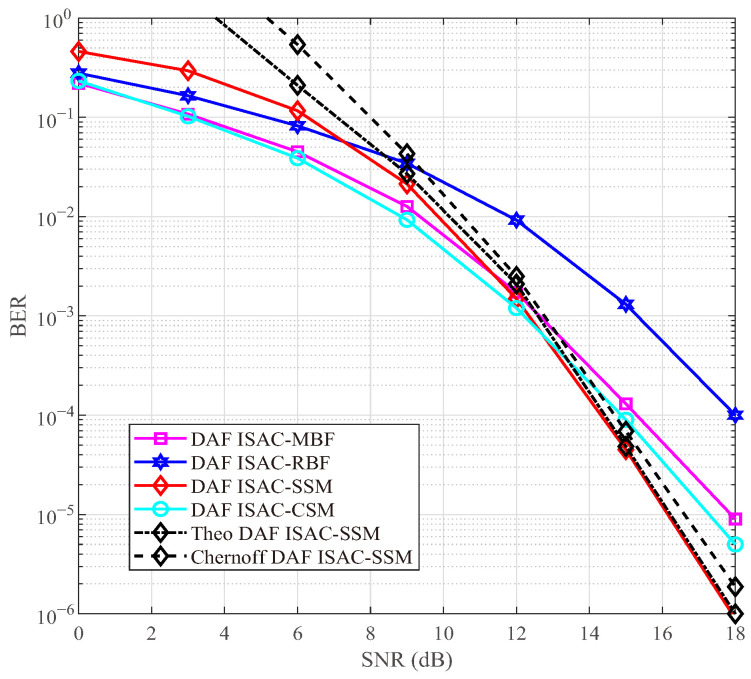
BER comparisons under 2-hop network with I=12.

In Figure 4 and Figure 5, we compare the BER performance among DAF ISAC-SSM, DAF ISAC-CSM, DAF ISAC-MBF, and DAF ISAC-RBF in a two-hop network with a time-invariant scenario, having *I* = 6 and 12 scattering clusters, respectively. In Figure 4 and Figure 5, $I_{s}=4$ clusters with the highest gains are selected as candidate clusters. All the mentioned schemes use ISAC radar probing to select RV and adopt the DAF strategy at the RV. For the sake of fairness, SE is used as the evaluation metric. To achieve an SE of 4 bps/Hz, the proposed DAF ISAC-SSM scheme employs QPSK, while the DAF ISAC-MBF and DAF ISAC-RBF scheme use 16-QAM. The DAF ISAC-CSM scheme uses binary phase shift keying with the 4 activated number of RF chains. Figure 4 and Figure 5 illustrates that under high SNR, the Monte Carlo simulation closely aligns with the theoretical simulation, and the Monte Carlo simulation results consistently fall below the Chernoff upper bound. Moreover, when the total number of scattering clusters is large, such as I=12, the proposed DAF ISAC-SSM scheme can achieve a coding gain of 1.5 dB, 2 dB, and 4 dB over DAF ISAC-CSM, DAF ISAC-MBF, and DAF ISAC-RBF, respectively. These advantages stem from a 50% index bit rate, which in turn enables the utilization of a lower modulation order. Although DAF ISAC-MBF steers its beam towards the scattering cluster with the maximum path gain, the increase in the number of scattering clusters augments the probability of acquiring $I_{s}=4$ candidate scattering clusters that possess relatively higher path gains. Therefore, DAF ISAC-SSM shows superior performance.

**Figure 6:**
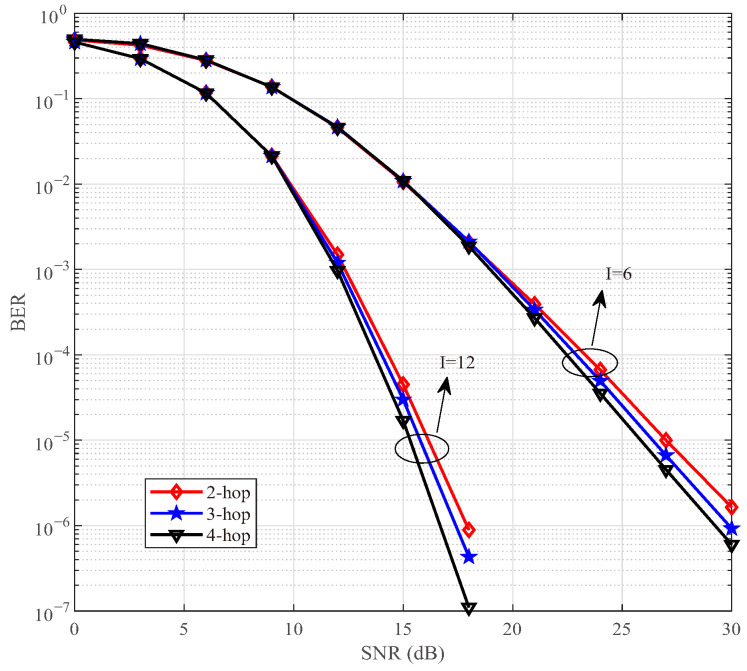
BER comparisons of DAF ISAC-SSM under different networking hops.

In Figure 6, we compare the BER performance of the proposed DAF ISAC-SSM under different networking hops with *I* = 6 and 12 scattering clusters. Simulations show that an increase in the number of scattering clusters enhances the reliability. Regarding a two-hop network, the scenario with *I* = 12 scattering clusters achieves a coding gain of approximately 10 dB relative to the situation where *I* = 6 at BER = $10^{-5}$. For the scenario with a fixed number of scattering clusters, it becomes clear that an augmentation in the number of network hops serves to strengthen the system’s robustness. In Figure 6 with *I* = 12, at BER = $10^{-6}$, the 4-hop network enjoys a coding gain of 0.5 dB and 1.5 dB over the two-hop network and the three-hop network, respectively. These improvements can be attributed to two factors. Firstly, during the relay forwarding stage, the DAF strategy adopts a detect-then-forward approach, which effectively mitigates the noise accumulation originating from the previous hop at the receiver end of each hop. Secondly, by leveraging the radar sensing capabilities, the RV is selected based on the principle of maximizing the link SNR. Consequently, it ensures the quality of the communication link and enhances the robustness of the network.

### 4.3. BER in Time-Variant Channel

**Figure 7:**
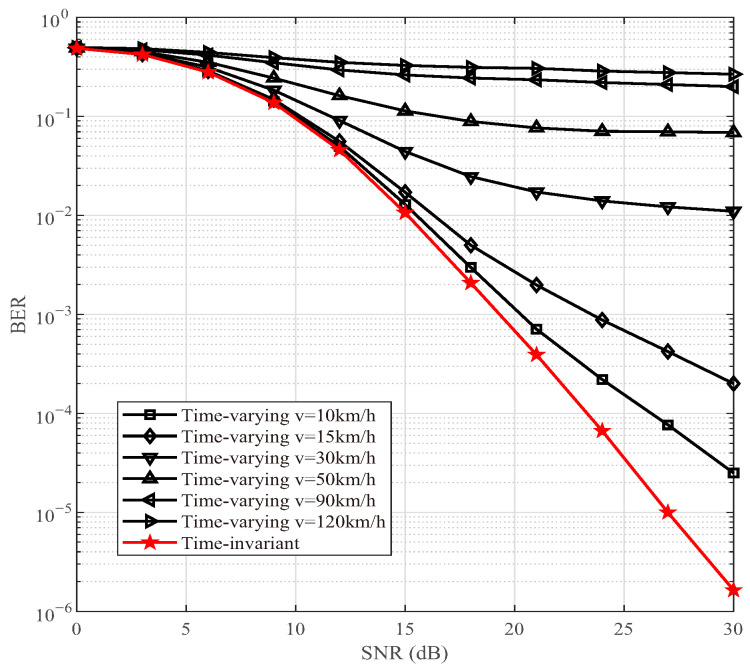
BER comparisons of DAF ISAC-SSM without compensation in time-varying channels.

**Figure 8:**
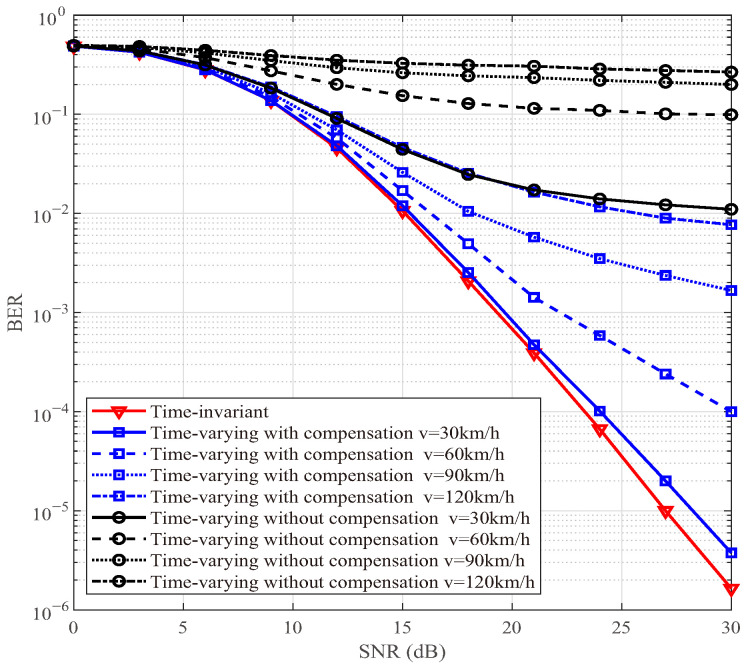
BER comparisons of DAF ISAC-SSM with compensation in time-varying channels.

**Figure 9:**
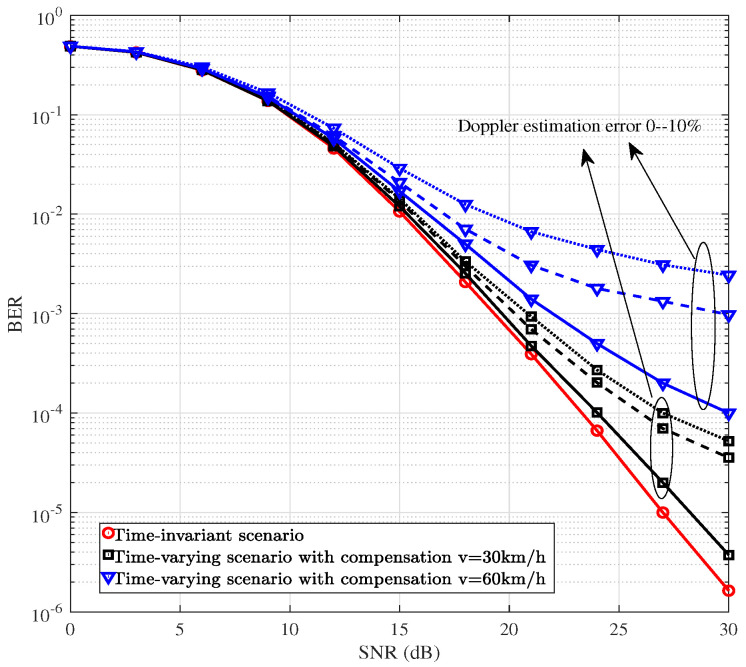
BER comparisons of DAF ISAC-SSM with different Doppler estimation error in time-varying channels.

We then investigate whether the BER advantage remains valid in time-varying channels, as depicted in Figure 7, Figure 8 and Figure 9. Without loss of generality, DAF ISAC-SSM is set as *I* = 6 and $I_{s}$ = 4 with 4-QAM modulation similar to Figure 4. Building upon the time-varying V2V communication scenarios expounded in Table 6 [35,36], simulations are executed for the four previously mentioned scenarios, i.e, Urban micro-unit, Suburban, Urban road, and Highway. In our simulations, four distinct V2V scenarios are evaluated under representative speed conditions to accurately reflect real-world operational environments, as shown in the fourth column of Table 6. Specifically, vehicle speeds are set to 10 km/h for Urban micro-unit environments such as high-density vehicle networks near intersections with frequent blocking, relevant for collision avoidance systems, 15 km/h for Suburban settings reflecting calm residential traffic, and two discrete values of 30 km/h and 50 km/h for Urban roads capturing both congested and free-flowing arterial conditions. For Highway scenarios, two high-speed cases of 90 km/h and 120 km/h are used to assess performance under common cruising and extreme high-velocity conditions, respectively, where pronounced Doppler effects and stringent latency demands are critical. These chosen speeds ensure comprehensive coverage of typical V2V use cases and facilitate reproducible analysis of communication reliability and Doppler tolerance. The normalized Doppler frequency shifts corresponding to the respective speeds are 0.006, 0.009, 0.018, 0.03, 0.053, and 0.07. In Figure 7, within a relatively low-SNR regime, the gap between the time-varying scenario and the ideal benchmark is minimized. Nevertheless, in the high-SNR region, as $v_{m}$ increases, the performance of the time-varying scenario deteriorates precipitously, presenting a floor effect.

Figure 8 depicts the BER performances of the DAF ISAC-SSM scheme across the time-invariant scenario, the time-varying scenario without Doppler compensation, and the time-varying scenario with Doppler compensation. For the time varying scenario, four different levels of maximum Doppler shift, namely 0.018, 0.035, 0.053, and 0.07 are considered. These values correspond to *v* = 30 km/h, 60 km/h, 90 km/h, and 120 km/h, respectively. The performance of DAF ISAC-SSM in time-invariant channel serves as the ideal benchmark for comparison. The superior performance of the Doppler-compensated scenario, particularly at a high SNR, demonstrates that the radar-derived angle information is instrumental in estimating and pre-compensating for the Doppler shift. This shift is the primary cause of time-selective fading in the V2V channel. Therefore, even coarse sensing data directly enables the mitigation of time selectivity, which is crucial for reliable high-speed communication.

Recall that in the previous simulations, we assumed perfect Doppler estimation. To assess the impact of Doppler estimation error on performance degradation, we introduce errors and conduct simulations. Figure 9 illustrates the Doppler compensation error performance for a two-hop network with 6 scattering clusters. In the Doppler compensation error model given by Equation (19), $e_{n,l}$ represents the error term, which follows a Gaussian distribution with zero mean and variance $\sigma_{w}^{2}=\eta N_{0}$. Here, η is a positive proportional coefficient associated with the angle error arising from imperfect radar detection. As can be observed from Figure 9, the performance of DAF ISAC-SSM with a η=5% estimation error closely approximates that of the ideal estimation. When the error is η=10% and the BER is $10^{-4}$, there is only a 2-dB loss for v=30 km/h. These results suggest that DAF ISAC-SSM maintains a high degree of reliability even in the presence of Doppler compensation errors.

### 4.4. Complexity Analysis

**Figure 10:**
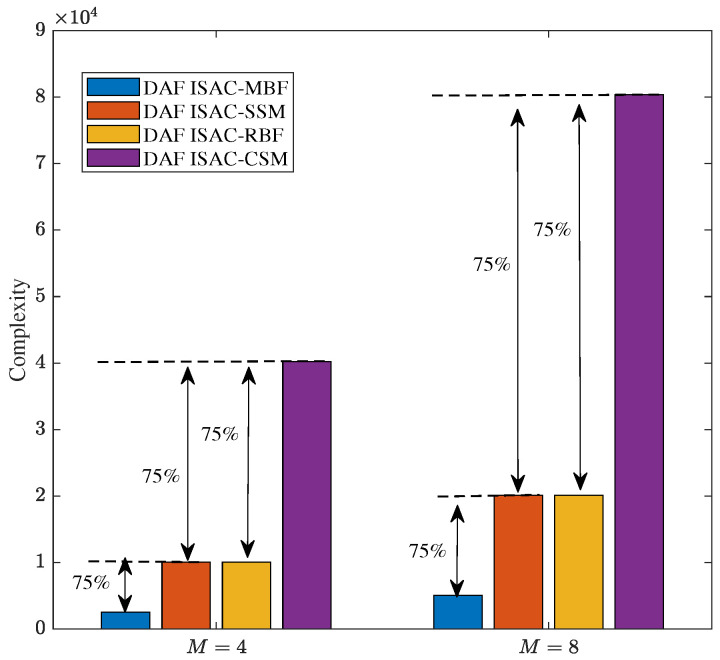
Complexity comparison of different schemes with M=4 and M=8.

To further validate the effectiveness of the proposed scheme, we present a complexity analysis of the proposed DAF ISAC-SSM over the three mentioned comparison schemes. Building upon Equation (18), we are able to derive the number of real-valued multiplications within the DAF ISAC-SSM system. Subsequently, through a comprehensive comparative analysis, we determine the real-valued multiplications associated with DAF ISAC-MBF, DAF ISAC-RBF, and DAF ISAC-CSM. The specific complexity analysis, expressed in terms of real-valued multiplications, for these diverse ISAC-V2V systems are presented in Table 7. Furthermore, Figure 10 illustrates the specific complexity of the four schemes under two modulation orders, i.e., *M* = 4 and *M* = 8.

As can be seen from Figure 10, the complexity of our proposed DAF ISAC-SSM scheme is nearly equivalent to that of ISAC-RBF. In contrast, when compared with the DAF ISAC-CSM scheme, it shows a 75% reduction in complexity. Meanwhile, relative to the DAF ISAC-MBF scheme, the DAF ISAC-SSM scheme exhibits a 75% increase in complexity. The reason for these complexity differences lies in the operational mechanisms of each scheme. The DAF ISAC-MBF receiver can ascertain the transmission direction without the need to explore candidate scatter clusters, allowing the lowest detection complexity. Thus, our proposed scheme makes a trade-off by sacrificing a portion of complexity in pursuit of enhanced reliability. For both DAF ISAC-SSM and DAF ISAC-RBF, which select from four candidate scatter clusters, they need to traverse these clusters to identify the cluster chosen by the transmitter. This traversal process increases their complexity. Regarding the DAF ISAC-CSM, it not only select four clusters but also employs two-dimensional vector symbols, resulting in the highest complexity.

In communication system design, it is essential to carefully balance the trade-offs among data rate, computational complexity, and communication reliability. As the modulation order *M* increases, the transmission rate is improved, but at the expense of increased system complexity and reduced reliability. Higher-order modulation schemes (e.g., from QPSK to 8-QAM) feature more densely spaced constellation points, which degrades BER performance under a given SNR. Moreover, the computational complexity of the ML detector grows linearly with *M*, i.e., O(M). Therefore, in practical system design, the selection of an appropriate *M* should be determined based on channel conditions, hardware processing capability, and quality-of-service requirements. For instance, a lower-order modulation may be chosen in low-SNR environments to ensure reliability, whereas higher-order modulation can be employed in favorable channel conditions to support higher data rates. Figure 4, Figure 5, Figure 6, Figure 7, Figure 8, Figure 9 and Figure 10 provide valuable design insights into this tradeoff by quantitatively evaluating the complexity and error performance under various configurations.

## 5. Conclusions

In this paper, we incorporate ISAC and SSM into multi-hop V2V networks to enhance system reliability. By effectively exploiting the sensed positioning information, we identify and select the vehicle with the maximum SNR as the relay vehicle. Concurrently, the employment of the DAF method serves as a crucial strategy to mitigate noise accumulation, thus enabling the proposal of the DAF ISAC-SSM system. Moreover, a meticulously designed simple first-order Doppler compensator is introduced to boost the robustness against Doppler effects. Through ML detection, we derive the CPEP and the corresponding BER upper bound expression. Theoretical analyses and numerical simulations are carried out to verify the advantages of DAF ISAC-SSM in terms of BER and EE. Specifically, the proposed DAF ISAC-SSM scheme can not only achieve a coding gain of 1.5 dB, 2 dB, and 4 dB over DAF ISAC-CSM, DAF ISAC-MBF, and DAF ISAC-RBF in a two-hop network with 12 scattering clusters but also exhibits robust performance with less than a 1.5 dB error degradation under 0.018 Doppler shifts. Given its outstanding performance in both time-invariant and time-varing channels, DAF ISAC-SSM emerges as a promising multi-hop V2V transmission scheme for future vehicular networks. Future work may focus on the following: (i) adaptive DAF ISAC-SSM index mapping for dynamic scattering clusters in real-world urban mobility; (ii) low-complexity detection algorithms for resource-constrained vehicular terminals; (iii) joint sensing–communication resource allocation under varying service priorities.

## Figures and Tables

**Table 1:** List of acronyms.

Acronym	Full Form
ISAC	Integrated Sensing and Communication
IM	Index Modulation
EE	Energy Efficiency
V2V	Vehicle-to-Vehicle
ISAC-SSM	Spatial Scattering Modulation based ISAC
DAF	Detect-Amplify-and-Forward
V2X	Vehicle-to-Everything
BER	Bit Error Rate
MIMO	Multiple-Input Multiple-Output
OFDM	Orthogonal Frequency Division Multiplexing
MAJoRCom	Multi-Carrier Agile Joint Radar Communication
CAESAR	Carrier Agile Phased Array Radar
FMCW	frequency modulated continuous wave
mmWave	Millimeter-Wave
SPIM	Spatial Path Index Modulation
SE	Spectral Efficiency
SV	Source Vehicle
RV	Relay Vehicle
TV	Target Vehicle
CPEP	Conditional Pairwise Error Probability
QAM	Quadrature Amplitude Modulation
COMM	Communication
CSM	Conventional Spatial Multiplexing
MBF	Maximum Beamforming
RBF	Random Beamforming

**Table 2:** Notations relating to DAF ISAC-SSM system.

Channel & Signal Notation	Definition
$H_{c,n,l,m}$	COMM Channle
$a_{t}\left(\theta_{n,l,i}^{t}\right)$	Tx $N_{c,t}$-dimensional array response
$a_{r}\left(\theta_{n,l,i}^{r}\right)$	Rx $N_{c,r}$-dimensional array response
$\gamma_{r,n,l}$	Radar Rx signal-to-noise ratio
$\gamma_{c,n,l}$	COMM Rx signal-to-noise ratio
$p_{n,l}$	COMM Tx beamforming
$y_{c,n,l,m}$	Rx Signal before RF chain
${\bar{y}}_{c,n,l,m}$	Rx Signal after RF chain
${\tilde{y}}_{c,n,l,m}$	Rx Signal after compensation

**Table 3:** An example of bit mapping with $b=\left[b_{1},b_{2},b_{3},b_{4}\right]=\left[0,0,0,0\right]$, *M* = 4.

$\left[b_{1},b_{2}\right]$	*x*	$\left[b_{3},b_{4}\right]$	Index Map	$p_{n,l}$
00	$\left(1+1j\right)/\sqrt{2}$	00	1	$a_{t}\left(\theta_{n,l,1}^{t}\right)$
01	$\left(1-1j\right)/\sqrt{2}$	01	2	$a_{t}\left(\theta_{n,l,2}^{t}\right)$
10	$\left(-1+1j\right)/\sqrt{2}$	10	3	$a_{t}\left(\theta_{n,l,3}^{t}\right)$
11	$\left(-1-1j\right)/\sqrt{2}$	11	4	$a_{t}\left(\theta_{n,l,4}^{t}\right)$

**Table 4:** The definition and value corresponding to the notation defined in Equation (24).

Notation	Definition	Value [31]
$P_{RF}$	RF chain	250 mW
$P_{A}$	Amplifier	20 mW
$P_{ps}$	Phase shifter	30 mW
$P_{sp}$	Power splitter	10 mW
$P_{co}$	Power combiner	10 mW

**Table 5:** The system parameters for numerical simulation.

Notation	Definition	Value [17,28]
$f_{c}$	COMM carrier frequency	28 GHz
$f_{r}$	Radar carrier frequency	24 GHz
$N_{c,t}$	COMM Tx antennas	12
$N_{c,r}$	COMM Rx antennas	12
$N_{r,t}$	Radar Tx antennas	12
$N_{r,r}$	Radar Rx antennas	12
$G_{t}$	Radar Tx antenna gain	8 dB
$G_{r}$	Radar Rx antenna gain	8 dB
Ω	Target’s cross-section	100 $m^{2}$
*I*	Total scattering clusters	6/12

**Table 6:** V2V communication scenarios [35,36].

Scenarios	Speed Range	Description	Adopted Speed
Urban micro-unit	0–15 km/h	Dense buildings	10 km/h
Suburban	0–20 km/h	Low-rise buildings	15 km/h
Urban road	0–60 km/h	Many buildings	30 km/h and 50 km/h
Highway	60–120 km/h	Sparse buildings	90 km/h and 120 km/h

**Table 7:** Complexity analysis of different schemes under varying modulations.

Scheme	Real-Valued Multiplications
DAF ISAC-SSM	$4N_{c,t}N_{c,r}MI_{s}+4N_{c,t}MI_{s}+4MI_{s}$
DAF ISAC-MBF	$4N_{c,t}N_{c,r}M+4N_{c,t}M+4M$
DAF ISAC-RBF	$4N_{c,t}N_{c,r}MI_{s}+4N_{c,t}MI_{s}+4MI_{s}$
DAF ISAC-CSM	$16N_{c,t}N_{c,r}MI_{s}+16N_{c,t}MI_{s}+16MI_{s}$

## Data Availability

The data presented in this study are available on request from the first author.

## Appendix A

When the scattering cluster index $g\in \{1,\cdots I_{s}\}$ is accurately detected, the bit error originates from symbol detection, that is, $g=\hat{g}$, $x\neq \hat{x}$. The beamforming is given by $p_{n,l}=a_{t}\left(\theta_{n,l,g}^{t}\right)$. From Equations (8) and (17), the received signal after the weights of phase shifters can be expressed as

(A1) $$\begin{array}{cc} {\bar{y}}_{c,n,l,m}\left(g\right) & =a_{r}{\left(\theta_{n,l,g}^{r}\right)}^{*}y_{c,n,l,m}\\ \; & =a_{r}{\left(\theta_{n,l,g}^{r}\right)}^{*}\;\left(A_{n,l}H_{c,n,l}p_{n,l}x+n_{n,l,m}\right)\\ \; & =A_{n,l}a_{r}{\left(\theta_{n,l,g}^{r}\right)}^{*}H_{c,n,l}a_{t}\left(\theta_{n,l,g}^{t}\right)x\;\;+\;\;a_{r}{\left(\theta_{n,l,g}^{r}\right)}^{*}n_{n,l,m}\\ \; & ={\bar{A}}_{n,l}\alpha_{n,l,g}x+a_{r}{\left(\theta_{n,l,g}^{r}\right)}^{*}n_{n,l,m}. \end{array}$$

For CPEP [17], we have

(A2) $$\begin{array}{c} \;\;\;\;\;\;P_{n}\left(\left\{g,x\right\}\to \left\{g,\hat{x}\right\}|\alpha_{n,l,1},\cdots ,\alpha_{n,l,I_{s}}\right)=P_{n}\left({\left|{\bar{y}}_{c,n,l,m}\left(g\right)-{\bar{A}}_{n,l}\alpha_{n,l,g}x\right|}^{2}\right.\\ \;\;\;\;\;\;\;\;\;\;\;\;\;\;\;\;\;\left.>{\left|{\bar{y}}_{c,n,l}\left(g\right)-{\bar{A}}_{n,l}\alpha_{n,l,g}\hat{x}\right|}^{2}\right) \end{array}.$$

Substituting Equation (A1) into Equation (A2), we can obtain

(A3) $$\begin{array}{c} \;P_{n}\left(\left\{g,x\right\}\to \left\{g,\hat{x}\right\}|\alpha_{n,l,1},\cdots ,\alpha_{n,l,I_{s}}\right)\\ =P_{n}\left({\left|a_{r}{\left(\theta_{n,l,g}^{r}\right)}^{*}n_{n,l,m}\right|}^{2}>\right.\\ \;\left.{\left|a_{r}{\left(\theta_{n,l,g}^{r}\right)}^{*}n_{n,l,m}+{\bar{A}}_{n,l}\alpha_{n,l,g}\left(x-\hat{x}\right)\right|}^{2}\right)\\ =P_{n}\left({\left|a_{r}{\left(\theta_{n,l,g}^{r}\right)}^{*}n_{n,l,m}\right|}^{2}>\right.\\ \;{\left|a_{r}{\left(\theta_{n,l,g}^{r}\right)}^{*}n_{n,l,m}\right|}^{2}+{\bar{A}}_{n,l}^{2}{\left|\alpha_{n,l,g}\left(x-\hat{x}\right)\right|}^{2}\\ \;\left.+2ℜ[a_{r}{\left(\theta_{n,l,g}^{r}\right)}^{*}n_{n,l,m}{\bar{A}}_{n,l}\alpha_{n,l,g}\left(x-\hat{x}\right)]\right)\\ =P_{n}\left(\;-({\bar{A}}_{n,l}^{2}{\left|\alpha_{n,l,g}\left(x-\hat{x}\right)\right|}^{2}-\right.\\ \;\left.2ℜ[{\bar{A}}_{n,l}a_{r}{\left(\theta_{n,l,g}^{r}\right)}^{*}n_{n,l,m}\alpha_{n,l,g}\left(x-\hat{x}\right)]>0)\right) \end{array},$$

define $\xi ={\bar{A}}_{n,l}^{2}{\left|\alpha_{n,l,g}\left(x-\hat{x}\right)\right|}^{2}+2ℜ\left[{\bar{A}}_{n,l}a_{r}{\left(\theta_{n,l,g}^{r}\right)}^{*}n_{n,l,m}\alpha_{n,l,g}\left(x-\hat{x}\right)\right]$; thus, we can obtain $\xi ∼N(\mu_{\xi },\sigma_{\xi }^{2})$, with $\mu_{\xi }={\bar{A}}_{n,l}^{2}{\left|\alpha_{n,l,g}\left(x-\hat{x}\right)\right|}^{2}$ and $\sigma_{\xi }^{2}=2N_{0}{\bar{A}}_{n,l}^{2}{\left|\alpha_{n,l,g}\right|}^{2}{\left|x-\hat{x}\right|}^{2}$. Therefore, we have

(A4) $$\begin{array}{cc} \;\;\;\;\;\;P_{n}\left(\left\{g,x\right\}\to \left\{g,\hat{x}\right\}|\alpha_{n,l,1},\cdots ,\alpha_{n,l,I_{s}}\right)\; & =Q\left(\sqrt{\frac{\mu_{\xi }^{2}}{\upsilon_{\xi }^{2}}}\right)\\ \; & =Q\left(\sqrt{\frac{{\bar{A}}_{n,l}^{2}{\left|\alpha_{n,l,g}\right|}^{2}{\left|\left(x-\hat{x}\right)\right|}^{2}}{2N_{0}}}\right), \end{array}$$

by applying Chernoff bound $Q\left(z\right)\leq \frac{1}{2}e^{\frac{-z^{2}}{2}}$ [37], the upper bound can be derived, and it is presented in Equation (27).

## Appendix B

When the scatter index detection $\hat{g}\in \left\{1,\cdots I_{s}\right\}$ is incorrect, i.e., $g\neq \hat{g}$, the symbol detection can be either correct, $x=\hat{x}$, or incorrect, $x\neq \hat{x}$, at this moment. As can be seen from Equation (8), the inner product of array responses with different directions is zero. Thus,

(A5) $$\begin{array}{cc} {\bar{y}}_{c,n,l,m}\left(\hat{g}\right) & =a_{r}{\left(\theta_{n,l,\hat{g}}^{r}\right)}^{*}y_{c,n,l,m}\\ \; & =A_{n,l}a_{r}{\left(\theta_{n,l,\hat{g}}^{r}\right)}^{*}H_{c,n,l}a_{t}\left(\theta_{n,l,g}^{t}\right)x+a_{r}{\left(\theta_{n,l,\hat{g}}^{r}\right)}^{*}n_{n,l,m}\\ \; & =a_{r}{\left(\theta_{n,l,\hat{g}}^{r}\right)}^{*}n_{n,l,m}. \end{array}$$

Then CPEP can be expressed as

(A6) $$\begin{array}{c} \;P_{n}\left(\left\{g,x\right\}\to \left\{\hat{g},\hat{x}\right\}|\alpha_{n,l,1},\cdots ,\alpha_{n,l,I_{s}}\right)\\ =P_{n}\left({\left|{\bar{y}}_{c,n,l,m}\left(g\right)-{\bar{A}}_{n,l}\alpha_{n,l,g}x\right|}^{2}\right.\\ \;\left.>{\left|{\bar{y}}_{c,n,l,m}\left(\hat{g}\right)-{\bar{A}}_{n,l}\alpha_{n,l,\hat{g}}\hat{x}\right|}^{2}\right)\\ =P_{n}\left({\left|a_{r}{\left(\theta_{n,l,g}^{r}\right)}^{*}n_{n,l,m}\right|}^{2}\right.\\ \;\left.>{\left|a_{r}{\left(\theta_{n,l,\hat{g}}^{r}\right)}^{*}n_{n,l,m}-{\bar{A}}_{n,l}\alpha_{n,l,\hat{g}}\hat{x}\right|}^{2}\right) \end{array},$$

define $\xi_{1}={\left|a_{r}{\left(\theta_{n,l,g}^{r}\right)}^{*}n_{n,l,m}\right|}^{2}$ and $\xi_{2}={\left|a_{r}{\left(\theta_{n,l,\hat{g}}^{r}\right)}^{*}n_{n,l,m}-{\bar{A}}_{n,l}\alpha_{n,l,\hat{g}}\left(\hat{x}\right)\right|}^{2}$. Because $a_{r}(\theta_{n,l,g}^{r}{)}^{*}n_{n,l,m}∼CN\left(0,N_{0}\right)$, $\frac{\xi_{1}}{N_{0}/2}$ is a chi-square random variable with two degrees of freedom, it follows an exponential distribution with a rate of 1/2. Regarding $\xi_{2}$, since $a_{r}{\left(\theta_{n,l,\hat{g}}^{r}\right)}^{*}n_{n,l,m}-{\bar{A}}_{n,l}\alpha_{n,l,\hat{g}}\hat{x}∼CN\left(-{\bar{A}}_{n,l}\alpha_{n,l,\hat{g}}\hat{x},N_{0}\right)$, $\frac{\xi_{2}}{N_{0}/2}$ is a non-central chi-square random variable with two degrees of freedom, and its non-central parameter is $\lambda_{\xi_{2}}=\frac{2{{\bar{A}}_{n,l}}^{2}{\left|\alpha_{n,l,\hat{g}}\right|}^{2}{\left|\hat{x}\right|}^{2}}{N_{0}}$ [17].

According to [38], the central chi-square distribution $\frac{\xi_{1}}{N_{0}/2}$ can be represented as

(A7) $$f\left(\frac{\xi_{1}}{N_{0}/2}\right)=\frac{1}{2}e^{-\frac{\xi_{1}}{N_{0}/4}}.$$

Thus, Equation (A6) can be calculated as

(A8) $$\begin{array}{cc} P_{n} & =\frac{1}{2}\int_{0}^{\infty }f(\frac{\xi_{2}}{N_{0}/2})\left(\int_{\frac{\xi_{2}}{N_{0}/2}}^{\infty }e^{-\frac{\xi_{1}}{N_{0}/4}}d\frac{\xi_{1}}{N_{0}/2}\right)d\frac{\xi_{2}}{N_{0}/2}\\ \; & =\int_{0}^{\infty }f\left(\frac{\xi_{2}}{N_{0}/2}\right)e^{-\frac{\xi_{2}}{N_{0}/4}}d\frac{\xi_{2}}{N_{0}/2}. \end{array}$$

It can be seen that Equation (A8) satisfies the form of the moment generating function (MGF) [39]. Accordingly, the MGF of a non-central chi-square distribution with two degrees of freedom can be represented as

(A9) $$M_{s}\left(\frac{\xi_{2}}{N_{0}/2}\right)=\left(\frac{1}{1-2z\sigma^{2}}\right)e^{\frac{z\mu^{2}}{1-2z\sigma^{2}}}.$$

Substituting Equation (A9) into Equation (A8), we have

(A10) $$\begin{array}{c} P_{n}\left(\left\{g,x\right\}\to \left\{\hat{g},\hat{x}\right\}|\alpha_{n,l,1},\cdots ,\alpha_{n,l,I_{s}}\right)=\left(\frac{1}{1+\sigma^{2}}\right)e^{-\frac{\mu^{2}}{2(1+\sigma^{2})}}. \end{array}.$$

For $\frac{\xi_{2}}{N_{0}/2}$, given that $\sigma^{2}=N_{0}$ and $\mu =-{\bar{A}}_{n,l}\alpha_{n,l,\hat{g}}\hat{x}$, the CPEP under the condition of incorrect scatter index detection can be expressed as shown in Equation (28).

## References

[1] Chen R., Sun S., Liu Y., Hu X., Hui Y., Cheng N. (2024). Proactive effects of C-V2X-based vehicle-infrastructure cooperation on the stability of heterogeneous traffic flow. IEEE Internet Things J..

[2] Chen S., Hu J., Shi Y., Zhao L., Li W. (2020). A vision of C-V2X: Technologies, field testing, and challenges with Chinese development. IEEE Internet Things J..

[3] Liu L., Wang Z., Zhang C. (2024). Design and selection of government regulations for vehicle supply chains: A chinese perspective. Comput. Ind. Eng..

[4] Choi J., Va V., Gonzalez-Prelcic N., Daniels R., Bhat C.R., Heath R.W. (2016). Millimeter-wave vehicular communication to support massive automotive sensing. IEEE Commun. Mag..

[5] Cheng X., Zhang H., Yang Z., Huang Z., Li S., Yu A. (2022). Integrated sensing and communications for internet of vehicles: Current status and development trend. J. Commun..

[6] Mesleh R.Y., Haas H., Sinanovic S., Ahn C.W., Yun S. (2008). Spatial modulation. IEEE Trans. Veh. Technol..

[7] Basar E., Aygolu U., Panayırcı E., Poor H.V. (2013). Orthogonal frequency division multiplexing with index modulation. IEEE Trans. Signal Process..

[8] Xu Z., Petropulu A., Sun S. A joint design of MIMO-OFDM dual-function radar communication system using generalized spatial modulation. Proceedings of the 2020 IEEE Radar Conference (RadarConf20).

[9] Ma D., Shlezinger N., Huang T., Shavit Y., Namer M., Liu Y., Eldar Y.C. (2021). Spatial modulation for joint radar-communications systems: Design, analysis, and hardware prototype. IEEE Trans. Veh. Technol..

[10] Xu J., Wang X., Aboutanios E., Cui G. (2023). Hybrid index modulation for dual-functional radar communications systems. IEEE Trans. Veh. Technol..

[11] Huang T., Shlezinger N., Xu X., Liu Y., Eldar Y.C. (2020). MAJoRCom: A dual-function radar communication system using index modulation. IEEE Trans. Signal Process..

[12] Ma D., Shlezinger N., Huang T., Liu Y., Eldar Y.C. (2021). FRaC: FMCW-based joint radar-communications system via index modulation. IEEE J. Sel. Top. Signal Process..

[13] Huang G., Ding Y., Ouyang S., Fusco V.F. (2021). Index modulation for OFDM RadCom systems. J. Eng..

[14] Hawkins H., Xu C., Yang L.-L., Hanzo L. (2024). IM-OFDM ISAC outperforms OFDM ISAC by combining multiple sensing observations. IEEE Open J. Veh. Technol..

[15] Yang Z., Gao S., Cheng X., Yang L. (2024). Superposed IM-OFDM (S-IM-OFDM): An enhanced OFDM for integrated sensing and communications. IEEE Trans. Veh. Technol..

[16] Wang J., He L., Song J. (2019). Towards higher spectral efficiency: Spatial path index modulation improves millimeter-wave hybrid beamforming. IEEE J. Sel. Top. Signal Process..

[17] Ding Y., Kim K.J., Koike-Akino T., Pajovic M., Wang P., Orlik P. (2017). Spatial scattering modulation for uplink millimeter-wave systems. IEEE Commun. Lett..

[18] Elbir A.M., Mishra K.V., Celik A., Eltawil A.M. Millimeter-wave radar beamforming with spatial path index modulation communications. Proceedings of the 2023 IEEE Radar Conference (RadarConf23).

[19] Elbir A.M., Mishra K.V., Abdallah A., Celik A., Eltawil A.M. (2024). Spatial path index modulation in mmwave/THz band integrated sensing and communications. IEEE Trans. Wirel. Commun..

[20] Liu J., Yang W., Zhang J., Yang C. (2020). Detecting false messages in vehicular ad hoc networks based on a traffic flow model. Int. J. Distrib. Sens. Netw..

[21] Wang H., Yang W., Wei W. (2024). Efficient algorithms for urban vehicular ad hoc networks quality based on average network flows. Peer Peer Netw. Appl..

[22] Gupta A., Sellathurai M., Ratnarajah T. (2023). End-to-end learning-based full-duplex amplify-and-forward relay networks. IEEE Trans. Commun..

[23] Arzykulov S., Nauryzbayev G., Tsiftsis T.A., Maham B., Abdallah M. (2019). On the outage of underlay CR-NOMA networks with detect-and-forward relaying. IEEE Trans. Cogn. Commun. Netw..

[24] Domanovitz E., Khisti A., Tan W.-T., Zhu X., Apostolopoulos J. (2022). State-dependent symbol-wise decode and forward codes over multihop relay networks. IEEE Trans. Inf. Theory.

[25] Guo X., Liu C., Bao J., Jiang B. (2022). Multi relay selection and power allocation of low complexity amplify and forward cooperation. Acta Electron. Sin..

[26] Yu R., Wang P. A reliable intra-relay cooperative relay network coupling with spatial modulation for the dynamic V2V communication. Proceedings of the 2022 7th International Conference on Communication, Image and Signal Processing (CCISP).

[27] Ayach O.E., Rajagopal S., Abu-Surra S., Pi Z., Heath R.W. (2014). Spatially Sparse Precoding in Millimeter Wave MIMO Systems. IEEE Trans. Wirel. Commun..

[28] Fan Y., Gao S., Duan D., Cheng X., Yang L. (2023). Radar integrated MIMO communications for multi-hop V2V networking. IEEE Wirel. Commun. Lett..

[29] Barton D. (2012). Radar Equations for Modern Radar.

[30] (2021). IEEE Standard for Wireless Access in Vehicular Environments (WAVE)–Networking Services—Redline.

[31] Gao X., Dai L., Sayeed A.M. (2018). Low RF-complexity technologies to enable millimeter-wave MIMO with large antenna array for 5G wireless communications. IEEE Commun. Mag..

[32] Salameh H.A.B., Mahdawi L., Musa A., Hailat T.F. (2020). End-to-end performance analysis with decode-and-forward relays in multihop wireless systems over *α*–*η*–*μ* fading channels. IEEE Syst. J..

[33] Zhu X., Chen W., Li Z., Wu Q., Zhang Z., Wang K., Li J. On the performance of RIS-aided spatial scattering modulation for mm wave transmission. Proceedings of the GLOBECOM 2023—2023 IEEE Global Communications Conference.

[34] Zhu X., Chen W., Wu Q., Li J., Cheng N., Chen F., Li C. (2024). Performance analysis of RIS-aided double spatial scattering modulation for mmwave mimo systems. IEEE Trans. Wirel. Commun..

[35] Liu Y., Zhang M., Wang H., Cheng X. Spatial modulation orthogonal frequency division multiplexing with subcarrier index modulation for V2X communications. Proceedings of the 2016 International Conference on Computing, Networking and Communications (ICNC).

[36] Cheng X., Li Y., Ai B., Yin X., Wang Q. (2015). Device-to-device channel measurements and models: A survey. IET Commun..

[37] Bucklew J., Sadowsky J. (1993). A contribution to the theory of chernoff bounds. IEEE Trans. Inf. Theory.

[38] Sengijpta S.K. (1994). Fundamentals of statistical signal processing: Estimation theory. Control Eng. Pract..

[39] Simon M.K. (2006). Probability Distributions Involving Gaussian Random Variables a Handbook for Engineers and Scientists.

